# Dynamic characteristics and functional analysis provide new insights into the role of *GauERF105* for resistance against *Verticillium dahliae* in cotton

**DOI:** 10.1186/s12870-023-04455-w

**Published:** 2023-10-18

**Authors:** Yanqing Wang, Muhammad Jawad Umer, Xiaoyan Cai, Mengying Yang, Yuqing Hou, Yanchao Xu, Raufa Batool, Teame Gereziher Mehari, Jie Zheng, Yuhong Wang, Heng Wang, Zhikun Li, Zhongli Zhou, Fang Liu

**Affiliations:** 1 National Key Laboratory of Cotton Bio-Breeding and Integrated Utilization/Institute of Cotton Research, Chinese Academy of Agricultural Sciences (ICR, CAAS), Anyang, Henan 455000 China; 2 College of Agronomy, Hebei Agricultural University/North China Key Laboratory for Crop Germplasm Resources of Ministry of Education, Baoding, 071001 Hebei China; 3 National Nanfan Research Institute of Chinese Academy of Agriculture Sciences, Sanya, 572025 China; 4 School of Agricultural Sciences, Zhengzhou University, Zhengzhou, 450001 Henan China; 5 State Key Laboratory for Biology of Plant Diseases and Insect Pest, Institute of Plant Protection, Chinese Academy of Agricultural Sciences, Beijing, 100000 China; 6 Ethiopian Institute of Agricultural Research, Mekhoni Agricultural Research Center, P.O BOX 47, Mekhoni, Tigray Ethiopia

**Keywords:** Verticillium wilt, Cotton, ERF, VIGS, Overexpression

## Abstract

**Background:**

The cotton industry suffers significant yield losses annually due to Verticillium wilt, which is considered the most destructive disease affecting the crop. However, the precise mechanisms behind this disease in cotton remain largely unexplored.

**Methods:**

Our approach involved utilizing transcriptome data from *G. australe* which was exposed to *Verticillium dahliae* infection. From this data, we identified ethylene-responsive factors and further investigated their potential role in resistance through functional validations via Virus-induced gene silencing (VIGS) in cotton and overexpression in Arabidopsis.

**Results:**

A total of 23 ethylene response factors (ERFs) were identified and their expression was analyzed at different time intervals (24 h, 48 h, and 72 h post-inoculation). Among them, *GauERF105* was selected based on qRT-PCR expression analysis for further investigation. To demonstrate the significance of *GauERF105*, VIGS was utilized, revealing that suppressing *GauERF105* leads to more severe infections in cotton plants compared to the wild-type. Additionally, the silenced plants exhibited reduced lignin deposition in the stems compared to the WT plants, indicating that the silencing of *GauERF105* also impacts lignin content. The overexpression of *GauERF105* in Arabidopsis confirmed its pivotal role in conferring resistance against *Verticillium dahliae* infection. Our results suggest that WT possesses higher levels of the oxidative stress markers MDA and H_2_O_2_ as compared to the overexpressed lines. In contrast, the activities of the antioxidant enzymes SOD and POD were higher in the overexpressed lines compared to the WT. Furthermore, DAB and trypan staining of the overexpressed lines suggested a greater impact of the disease in the wild-type compared to the transgenic lines.

**Conclusions:**

Our findings provide confirmation that *GauERF105* is a crucial candidate in the defense mechanism of cotton against *Verticillium dahliae* invasion, and plays a pivotal role in this process. These results have the potential to facilitate the development of germplasm resistance in cotton.

## Background

Cotton is an economic crop, while Verticillium wilts severely restrict cotton production [1]. Cotton (Gossypium spp.; tribe Gossypieae, family Malvaceae) has a high economic value and wide geographical distribution. There are a total of 53 species of cotton [2], of which 46 diploid cotton species are divided into 8 genome groups of A, B, C, D, E, F, G, and K. The remaining 7 tetraploid cotton species (AD)_1_-(AD)_7_ belong to one allopolyploid genome [3–5]. The A, B, E, and F cotton-type genomes are distributed in Asia and Africa, the C, G, and K cotton-type genomes are distributed in Australia, and the D and AD cotton-type genomes are distributed in America.

Cotton Verticillium wilt is caused by the pathogen *Verticillium dahliae*, which can be further classified into *Verticillium dahliae* and *Verticillium albo-atrum*. *Verticillium dahliae* can cause the wilt of a variety of plants. *Verticillium dahliae* can infect the vascular bundles of the cotton plant. Verticillium wilt was first discovered in the United States in 1915, then introduced to China in 1935, and successively broke out in major cotton production areas [6]. In addition to cotton, fruits, and vegetables such as potatoes, tomatoes, grapes, and some woody plants can be attacked by *Verticillium dahliae* [7]. The main hosts of Verticillium black and white are alfalfa, hops, soybeans, tomatoes, potatoes, and some weeds [8, 9]. After investigations, the diseases in cotton fields in China are mainly caused by *Verticillium dahliae* [10].

Cotton Verticillium wilt is one of the soil-borne fungal vascular diseases, which leads to yearly yield losses of over 30% and a severe economic loss of roughly 250–310 million dollars for China [11]. Though, it is hard to control pathogenic harm to cotton plants, even though many attempts have been made, among them are the use of fungicides as well as cultural methods [12, 13]. Generally, to protect plants from pathogenic damage, disease-resistant cultivars must be widely planted. Although certain genes were characterized in cotton plant protection against pathogens, several effective candidate genes were updated for their use in disease-resistant breeding [11, 14, 15]. Thus, molecular mechanisms of plant resistance against *Verticillium dahliae* as well as the functional analysis of genes linked to defense must be explored deeply.

Among the signal pathways that plants respond to biological stress, the ethylene signaling pathway plays an important role, and many disease-resistant genes are induced and regulated by this signal pathway [16]. Overexpression of *AtERF1* directly activates the expression of plant defensins (PDF1.2) and improves plant resistance to pathogens [17, 18]. The T-DNA insertion mutant of *AtERF14* increases the susceptibility of Arabidopsis to *Fusarium oxysporum* infection [19]. *AtERF96* positively regulates the resistance of Arabidopsis to necrotic pathogens by enhancing the expression of *PDF1.2a*, *PR-3*, *PR-4*, and *ORA59* [20]. The overexpression of *GmERF5* in soybean increases its resistance to *Phytophthora sojae*, and it can positively regulate the expression of PR genes after being induced by *Phytophthora sojae* [21]. Overexpression of *ZmERF105* can increase the resistance of maize to *S. sphaerocephala*, while the *erf105* mutant strain showed the opposite phenotype; after *ZmERF105* overexpression strains were infected with *S. sphalacca*, *ZmPR1a, ZmPR2, ZmPR5, ZmPR10.1*. The expression of disease-related genes such as *ZmPR10.2* is enhanced, on the contrary, the expression of the *PR* gene is reduced in the *ERF105* mutant line [22]. Two *ERF* transcription factor members *EREB1* and *EREB2* from sea island cotton were cloned, and *Verticillium dahliae* can induce the expression of these two genes [23]. SSH method to enrich some differentially expressed genes related to a defense response and cloned the gene *GbERF1-like* from sea island cotton [24]. The study showed that the overexpression of *GbERF1-like* activated the synthesis of lignin-related genes, and enhanced cotton and pseudo-resistance of Arabidopsis to Verticillium wilt.

In this study, the *GauERF105* gene was cloned and characterized from *Gossypium australe*. Subsequently, the functional role of this gene was elucidated in cotton through Virus-induced gene silencing (VIGS) and overexpression experiments conducted in Arabidopsis, focusing on its response to the pathogenic challenge posed by *Verticillium dahliae*. Our study enhances the understanding of the molecular mechanisms governing cotton's resistance to Verticillium wilt and might help to discover new disease-resistance genes in wild cotton species. Additionally, this work identifies new genetic resources that can be utilized in cotton breeding programs to improve disease resistance.

## Results

### Cloning and sequence analysis of *GauERF105*

**Fig. 1:**
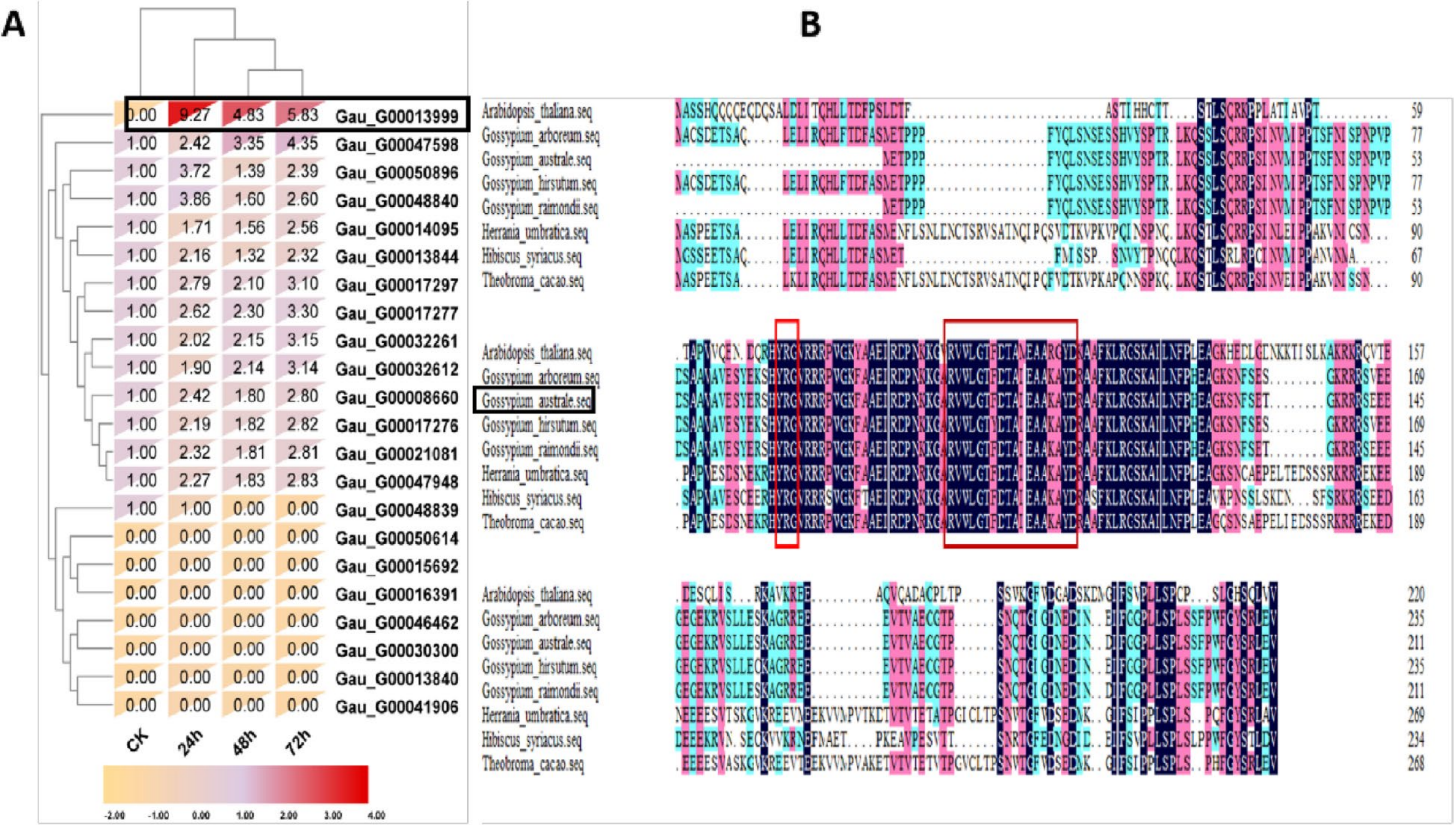
Expression and sequence analysis of *GauERF105 ***A** FPKM-based expression patterns of selected ERF genes. The red color represents the higher expressions in the heatmap. **B** Multiple sequence alignment of *GauERF105* in different plant species

By utilizing RNA-seq data previously published [25] for *G. australe* treated with *Verticillium dahliae*, we have successfully identified all of the *ERF* family genes. *GauERF105* was chosen for further analysis based on its high level of expression in response to attack by *Verticillium dahliae*, surpassing all other selected ERF genes **(**Fig. 1A**)**. The CDS sequence of *GauERF105* consisting of 639 bp was successfully cloned from the *G. australe*. This gene encodes 213 amino acids with a theoretical molecular mass of 23.609kD and an isoelectric point of 8.99. *GauERF105* was found to be located on chromosome 12 (10,153,228–10153866).

**Fig. 2:**
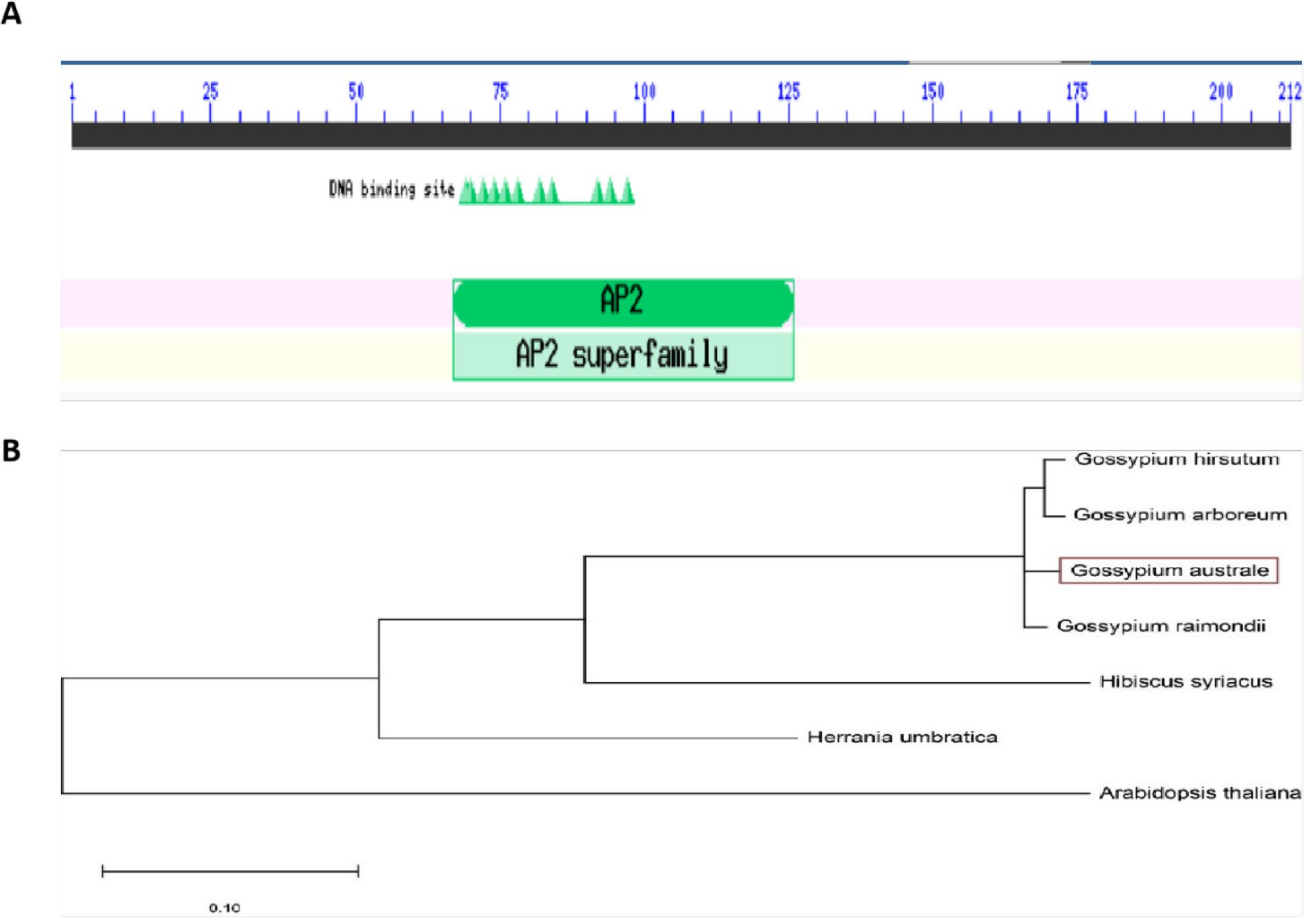
Conserved domain and phylogenetic analysis **A** Prediction of the conserved domain for *GauERF105*, **B** Phylogenetic analysis of *GauERF105*

Blastp search on NCBI and phylogenetic analysis revealed that *GauERF105* has a similarity to *Gossypium raimondii* (XP_01243908.1), *Gossypium arboretum* (XP_017636134.1), *Gossypium hirsutum* (XP_016721164.1), *Theobroma cacao* (XP_017873866.1), *Herrania umbratical* (XP_021286001.1), *Hibiscus syriacus* (XP_039051919.1) and *Arabidopsis thaliana* (AT5G51190), and the AP2/ERF domain contains conserved YRG and RAYD elements, which may play a key role in DNA binding and protein interactions (Fig. 2A). Using the CDD website [26] to predict the conserved domain of *GauERF105*, the results show that the amino acid sequence contains a typical AP2 domain at positions 58-125aa (Fig. 2B).

### Expression analysis of *GauERF105* under *Verticillium dahliae* stress

**Fig. 3:**
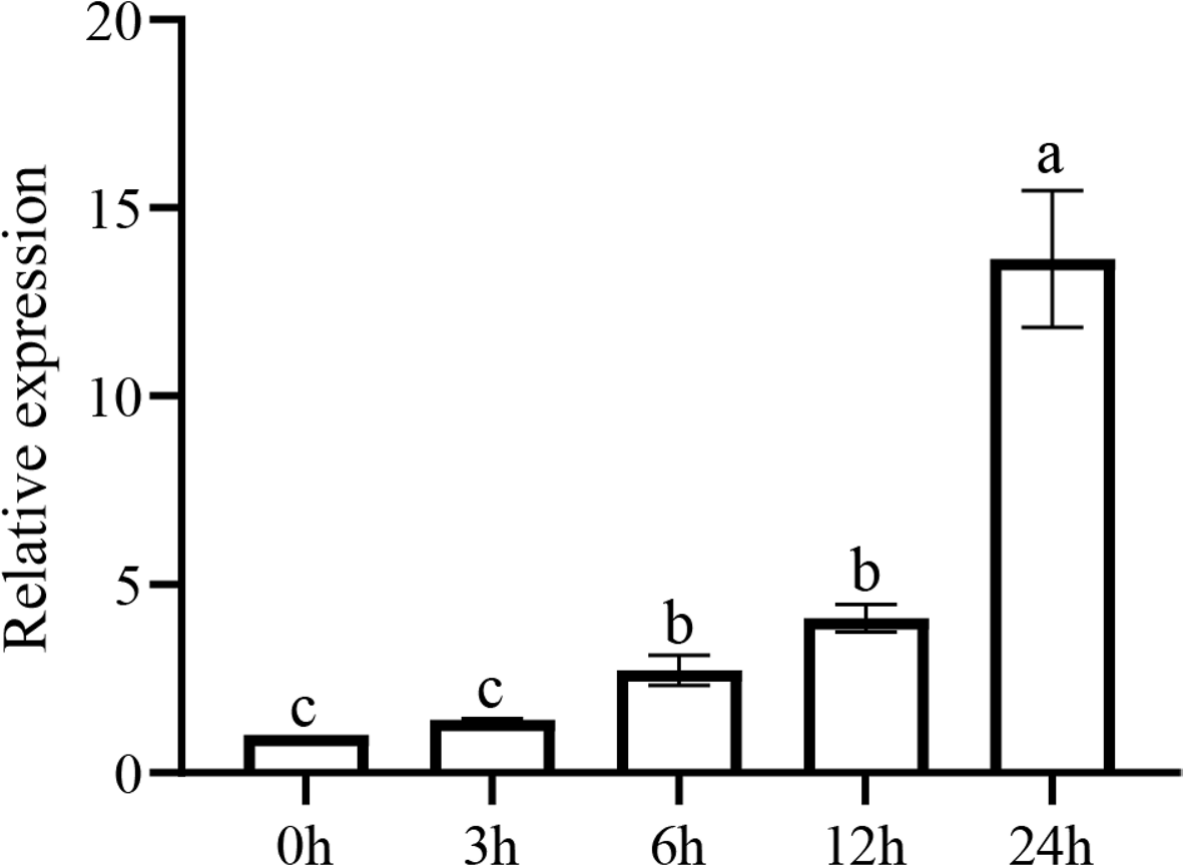
Expression pattern of *GauERF105* via RT-qPCR at 0, 3, 6, 12, and 24 h post-*Verticillium dahliae* inoculation

To confirm the involvement of *GauERF105* in the response to *Verticillium dahliae*, the expression patterns of this gene were analyzed in *Gossypium australe* leaves at 0 h, 3 h, 6 h, 12 h, and 24 h after fungal inoculation*.* It was observed that after inoculation, the expression level of *GauERF105* gradually increased from 0 to 24 h post-inoculation (Fig. 3). The highest gene expression was observed at 24 h post-inoculation. The results showed that *GauERF105* might be the key candidate involved in resistance against *Verticillium dahliae* infection.

### Overexpression of *GauERF105* enhances the resistance of Arabidopsis in response to Verticillium wilt attack

**Fig. 4:**
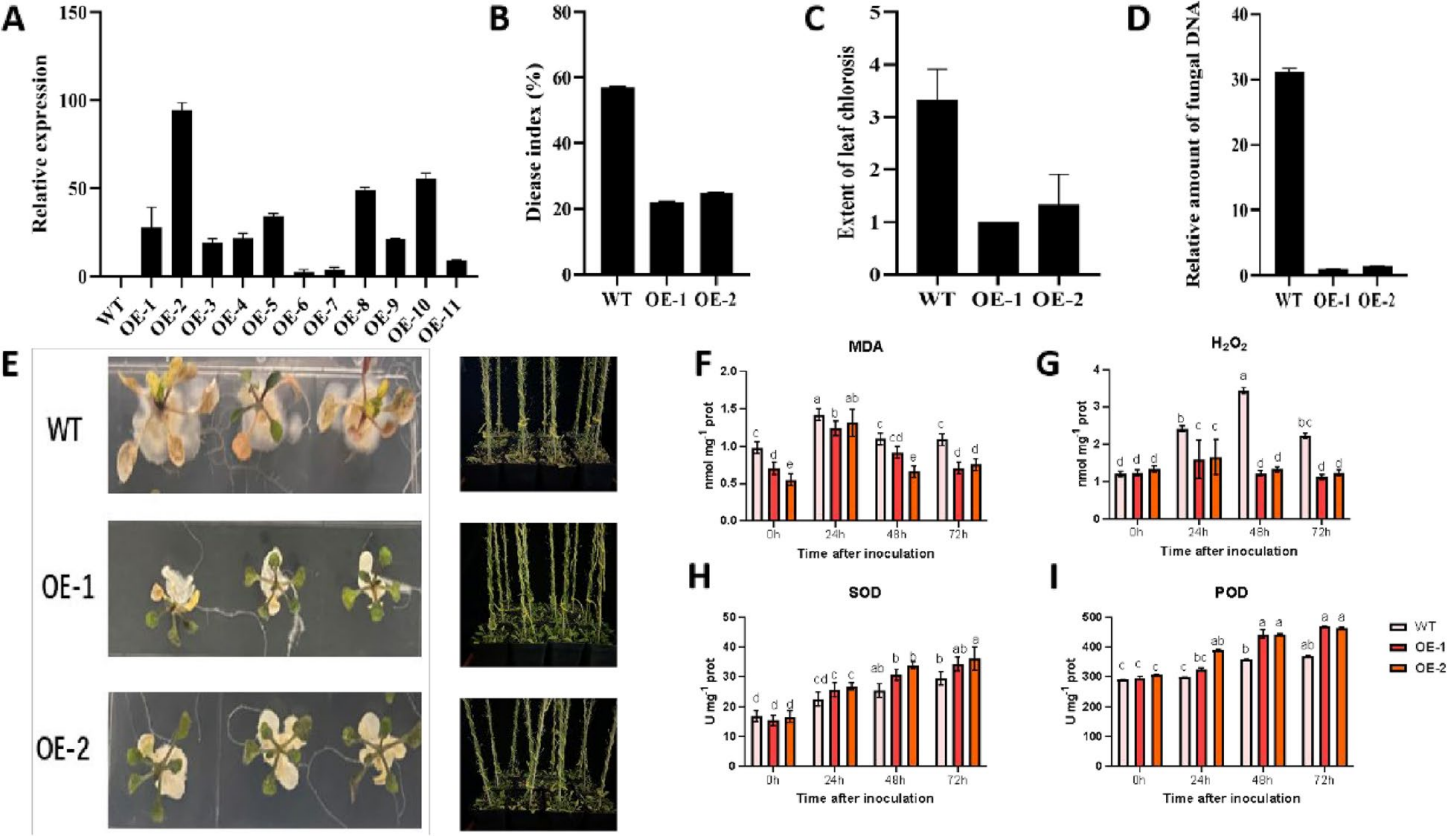
Overexpression of *GauERF105* enhances the resistance of Arabidopsis in response to Verticillium wilt attack. **A** Relative expression of *GauERF105* in the overexpressed lines, **B** Disease index in WT and overexpressed lines after fungal inoculation, **C** Extent of leaf chlorosis in wildtype and overexpressed lines after fungal inoculation, **D** Relative amount of fungal DNA in wildtype and overexpressed lines after fungal inoculation, **E** Impact of *Verticillium dahliae* infection of WT and overexpressed lines, **F** MDA content (a marker of oxidative damage) in wildtype and overexpressed lines after fungal inoculation, **G** H_2_O_2_ content (an indicator of oxidative stress) in wildtype and overexpressed lines after fungal inoculation, **H** SOD activity (a measure of antioxidant capacity) in wildtype and overexpressed lines after fungal inoculation, **I** POD activity (a measure of antioxidant capacity) in wildtype and overexpressed lines after fungal inoculation

To experimentally validate the involvement of *GauERF105* in the resistance to Verticillium wilt, we generated an overexpression vector, denoted as “*PBI121-GauERF105*”, and performed a transformation to introduce it into *Arabidopsis thaliana*. Positive seedlings were selected for screening on the MS solid medium containing kanamycin until the T_3_ generation where homozygous lines were screened. Samples from eleven positive T_3_ generation plants were collected to analyze the expression levels of *GauERF105*. Based on the results of RT-qPCR and agarose gel electrophoresis, we selected OE-1 and OE-2 for subsequent experiments (Fig. 4A).

An experiment was conducted to evaluate the effects of introducing the overexpression vector “*PBI121-GauERF105*” into *Arabidopsis thaliana* on resistance to *Verticillium dahliae*. Seeds from the OE-1 and OE-2 lines were spot-planted onto MS medium supplemented with a small amount of the pathogen, and the growth and development of the resulting plants were observed. Results indicated that WT showed a more sensitive phenotype to *Verticillium dahliae* as compared to the transgenic lines “OE-1 and OE-2”. In addition, the degree of WT plants' resistance towards Verticillium wilt was lower than that of transgenic lines, which indicates that the overexpression of the *GauERF105* gene enhances the plant's resistance to *Verticillium dahliae* infection. We also planted wild-type Arabidopsis and transgenic lines in nutrient soil and observed the symptoms after inoculation. After overexpressing the *GauERF105* gene in Arabidopsis, the plant's ability to resist Verticillium wilt was significantly enhanced, which is consistent with the phenotype of Arabidopsis grown on MS plates. The disease index was further counted by using the formula already mentioned in the methodology section, we observed results were consistent with that of phenotypic observations (Fig. 4B). The extent of leaf chlorosis was observed higher in overexpressed lines as compared to WT (Fig. 4C). Wildtype and overexpressed Arabidopsis seedlings were grown in MS media and were inoculated with *V. dahliae* to quantify the fungal DNA and impact of disease severity, results showed that WT plants were affected more as compared to overexpressed lines (Fig. 4D-E).

### Measurement of oxidative stress markers and antioxidant enzyme activities

Quantification of oxidative stress markers and antioxidant activities are crucial for estimating a plant's response to biotic and abiotic stresses. Leaf samples were collected at different time intervals (0 h, 24 h, 48 h and 72 h) post-fungal inoculation. In our study, we measured both the accumulation of oxidative stress markers and the activities of antioxidant enzymes in order to evaluate oxidative stress levels and antioxidant capacity in the wild-type and overexpression plants in response to *Verticillium dahliae* infection. Specifically, we quantified the content of malondialdehyde (MDA) as a marker of oxidative damage to lipids and overall oxidative stress, the content of hydrogen peroxide (H_2_O_2_) as an indication of oxidative stress levels, and the activity of peroxidase (POD) and superoxide dismutase (SOD) to assess the antioxidant capacity of wild type and mutant plants [27, 28]. Herein, our results suggest that WT possesses higher levels of the oxidative stress markers MDA and H_2_O_2_ as compared to the overexpressed lines **(**Fig. 4F-G**)**. In contrast, the activities of the antioxidant enzymes SOD and POD were higher in the overexpressed lines compared to the WT **(**Fig. 4H-I**)**. These results suggest that the overexpression of *GauERF105* might play a role in coping with *V. dahliae* infection by reducing the oxidative damage due to ROS, thus making the plants more resistant to Verticillium wilt.

### DAB and trypan blue staining

**Fig. 5:**
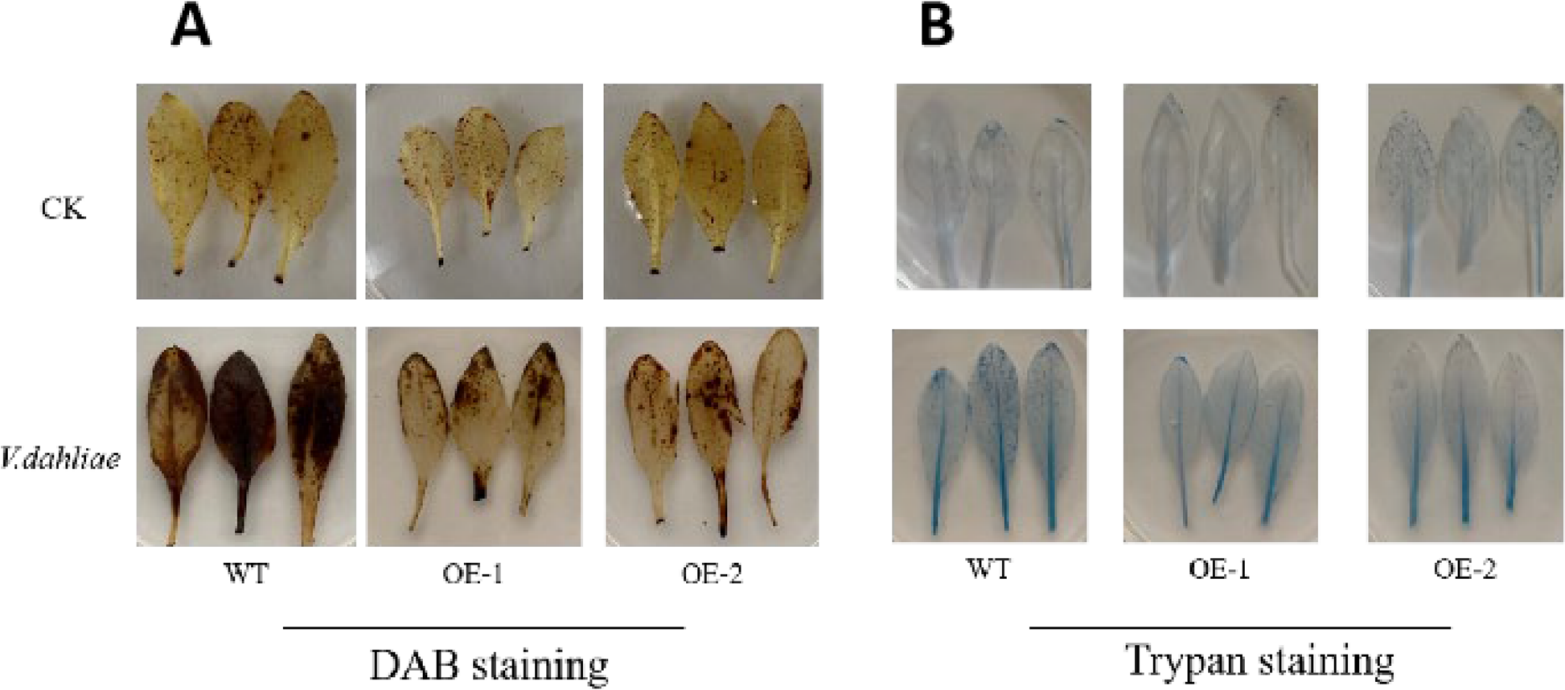
DAB and trypan blue staining **A** DAB staining to estimate the damage on Arabidopsis leaves after fungal inoculation, **B** DAB staining to estimate the damage on Arabidopsis leaves after fungal inoculation

Accumulation of reactive oxygen species (ROS) is an indication of oxidative damage caused by the stress of *Verticillium dahliae* infection. Following 72 h of inoculation, leaves were collected for DAB staining (Fig. 5A) to assess the level of ROS accumulation. Wild-type Arabidopsis and transgenic lines both started to accumulate ROS but the leaves of WT plants are affected more as compared to transgenic lines, indicating that the overexpression of *GauERF105* reduced the damage caused by *Verticillium dahliae* infection.

In response to *Verticillium dahliae* infection, the plants experienced significant damage leading to the production of a substantial number of dead cells. Therefore, the wild-type plants are stained darker. At the same time, the staining area of the wild-type *Arabidopsis thaliana* after inoculation was significantly larger than that of the transgenic *Arabidopsis thaliana*, which indicated that the damage of *Verticillium dahliae* to plants was greatly reduced after the *GauERF105* gene was overexpressed (Fig. 5B).

### Silencing of the *GauERF105* gene decreases the resistance against *Verticillium dahliae* in cotton

**Fig. 6:**
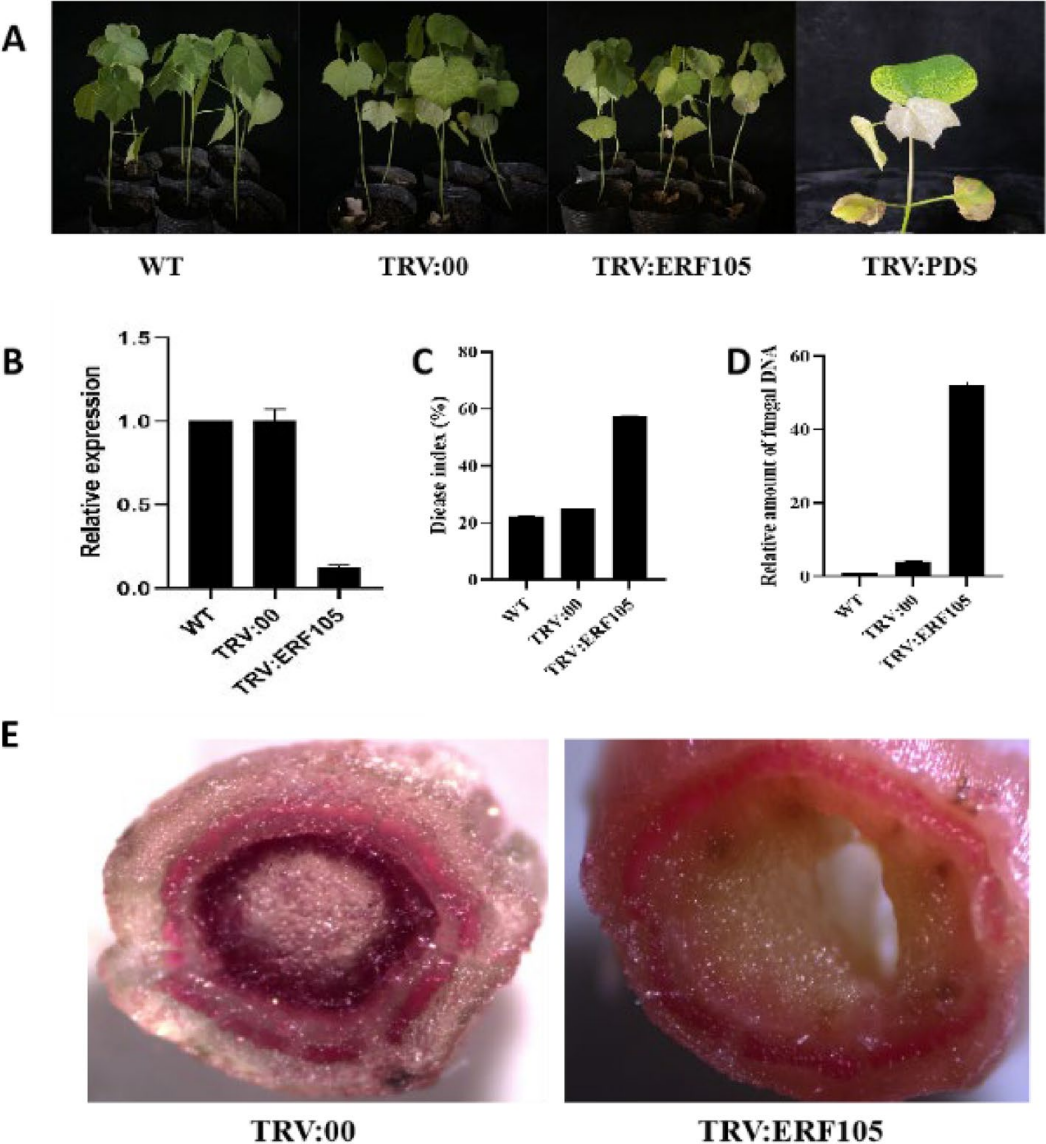
Silencing *GauERF105* decreases resistance against *Verticillium dahliae* in cotton **A** Representative images of WT, positive control, and VIGS plants, **B** Relative expression of *GauERF105* in WT, TRV:00, and TRV:*GauERF105*, **C** Disease index (%) in WT, TRV:00, and TRV:*GauERF105*, **D** Relative amount of fungal DNA in WT, TRV:00, and TRV:*GauERF105*, **E** Histochemical staining of cotton stem lignin. Bars show standard error. WT: Wild type, TRV:00: Positive control, TRV:*GauERF105*, the gene silenced plants

To establish the functional role of *GauERF105* in the response of cotton to *Verticillium dahliae* infection, we employed a technique known as virus-induced gene silencing (VIGS) to specifically downregulate the expression of *GauERF105* (Fig. 6). About 13 days after VIGS, the cotton leaves were injected with TRV: PDS bacteria, chlorosis started and an albino phenotype was observed, which proved that the VIGS system was established successfully and the results were accurate for the further experiment (Fig. 6A). The qRT-PCR results showed that the expression level of the *GauERF105* gene was significantly lower in silenced plants as compared to WT and TRV:00, indicating that the *GauERF105* gene is accurately silent .

Further, wild-type plants, empty vector plants, and silent plants were inoculated with *Verticillium dahliae*, and the phenotype was observed after 25 days of inoculation (Fig. 6B). Compared with the control plants, the leaves of the silent plants turned yellow, wilted and even fell off, and the disease index of the silent plants was also significantly higher. The degree of infection in silent plants was severe as compared to control plants (Fig. 6C). In addition, the leaves of WT, TRV:00, and TRV: *GauERF105* plants were quantified for *Verticillium dahliae*. The expression level of *Verticillium dahliae* in the silenced target gene plants was significantly higher than that of the control plants, which was consistent with the results of the previous disease and disease index investigation (Fig. 6D). We sterilized the cotton stems after inoculation and cultured them in a PDA solid medium. The infection rate of *Verticillium dahliae* in the TRV:00 plants was significantly lower than that of the TRV: *ERF105* plant. This indicates that silencing of the *GauERF105* gene weakens the plant's resistance to *Verticillium dahliae* infection and makes the plant more vulnerable to damage. Cotton lignin dying results showed that silent plants have inhibition of lignin as compared to wild-type and non-silent plants (Fig. 6E). Thus, proving the role of *GauERF105* in Verticillium wilt resistance in cotton.

### Expression of disease-resistant marker genes in cotton

**Fig. 7:**
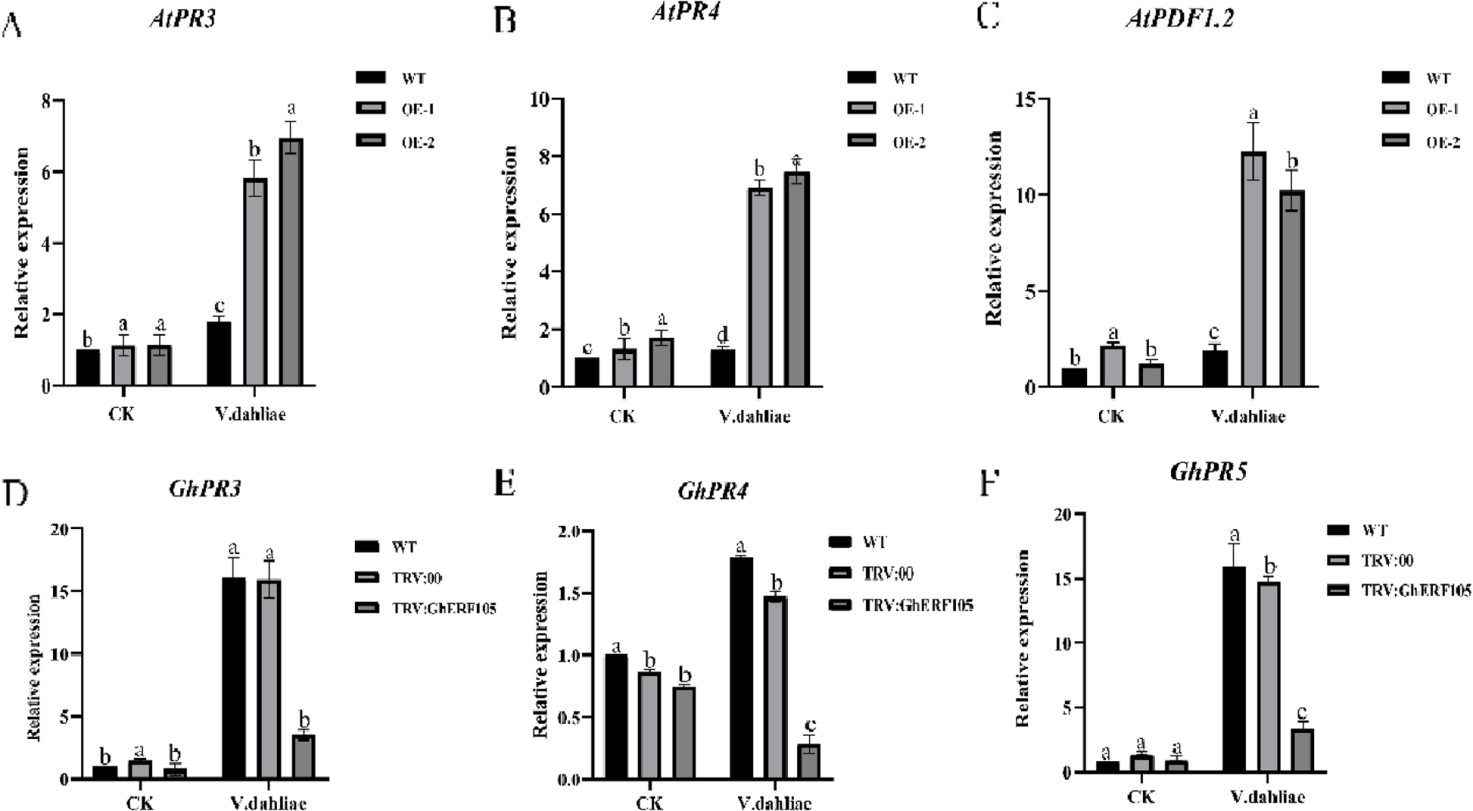
Expression analysis of disease resistance genes in transgenic Arabidopsis overexpressing *GauERF105* and cotton with *GauERF105* silenced via VIGS

In order to gain further insight into the regulatory role of the *GauERF105* gene in the plant disease resistance process, we conducted additional screening to assess the expression of several genes related to disease-resistant pathways in both Arabidopsis and cotton (Fig. 7A, B, C). The results showed that when the plants were inoculated with *Verticillium dahliae*, the expression of PRs increased; the expression of *AtPDF1.2*, *AtPR3,* and *AtPR4* genes in the two *GauERF105* gene-transformed transgene lines OE-1 and OE-2 lines was significantly higher than that in the wildtype. In VIGS plants, the expression of PRs genes in the silenced plants was significantly downregulated. It further illustrates that the *GauERF105* gene can activate hormone-related pathways to participate in plant disease resistance (Fig. 7D, E, F).

## Discussion

Following pathogen invasion, plants typically initiate a series of defense mechanisms as a means of protection. As one of the largest transcription factor families in plants, ERFs participate in the regulation of plant disease resistance.

ERF transcription factors are key regulators of plant defense mechanisms, activating the expression of genes related to resistance to pathogens and regulating the accumulation of secondary metabolites. These mechanisms enhance the plant's natural resistance to pests and diseases [29]. In the interaction network of plant immune response, SA and JA/ET have cross-effects [24]. Studies have shown that the SA pathway is involved in regulating the defense of plants against living vegetative pathogens, and JA and ET signal transduction are considered to be effective against pathogen attacks. Necrotrophic pathogens such as *B. cinerea* and *F. oxysporum* are more effective [30]. In this study, the *GauERF105* gene was screened and cloned from *G. australe*. Here we overexpressed *GauERF105* in Arabidopsis and checked the expression levels of AtPR3, AtPR4, and AtPDF1.2 genes in the transgenic lines OE-1 and OE-2. We observed that the expressions were significantly higher in transgene lines as compared to wild-type Arabidopsis, which shows that when the expression level of the *GauERF105* gene increases, the expression level of its downstream genes also increases; DAB staining results show that the staining degree of *GauERF105* in transgenic Arabidopsis leaves is lighter than that of wild-type Arabidopsis. This result shows that overexpression of the *GauERF105* gene reduces the oxidative damage of pathogens to plants and improves the resistance of Arabidopsis against Verticillium wilt. PR1 and PR5 are the downstream genes of the SA pathway, and PR3 and PR4 are the downstream genes of the ET/JA pathway. The promoter regions of these disease-related proteins have GCC-box. ERF transcription factors activate the expression of downstream defense genes by binding to GCC-box. So, as to enhance the plant's resistance to diseases and insect attacks. After overexpression of the potato *StERF94* transcription factor, the expression of PRs-related genes increased, thereby enhancing the potato's resistance to *Fusarium oxysporum* [31]. Overexpression of *VqERF112*, *VqERF114,* and *VqERF072* genes in Arabidopsis activated the SA signal-related genes *AtNPR1* and *AtPR1* and JA/ET signal-related genes *AtPDF1.2*, *AtLOX3*, *AtPR3*, and *AtPR4*, thereby enhancing the expression of Arabidopsis resistance to *Pst-DC3000* and *B. cinerea* [32].

In addition to the disease resistance phenotypes we observed, our biochemical analyses provided insights into the potential mechanisms by which GauERF105 overexpression enhances tolerance to *Verticillium dahliae*. We quantified the accumulation of oxidative stress markers like malondialdehyde (MDA) and hydrogen peroxide (H_2_O_2_), as well as the activities of antioxidant enzymes like superoxide dismutase (SOD) and peroxidase (POD). The overexpression lines showed lower MDA and H_2_O_2_ levels along with higher SOD and POD activities compared to wild-type plants, indicating that they experienced less oxidative damage and more robust antioxidant defenses during infection. These results suggest that *GauERF105* boosts resistance by modulating the plant's redox homeostasis and mitigating oxidative stress triggered by *V. dahliae*. The ability to scavenge ROS and prevent oxidative damage is crucial for plant defense against necrotrophic pathogens like *V. dahliae*. Our findings are in line with previous studies demonstrating the importance of antioxidant defenses in governing cotton's interaction with *V. dahliae* [33, 34]. The upregulation of antioxidant systems, controlled in part by defense-associated transcription factors like ERFs, is likely a key component of the enhanced tolerance we observed in the *GauERF105* overexpression lines. Our work provides evidence that *GauERF105* confers resistance by coordinating plant antioxidant defenses to counteract Verticillium-induced oxidative stress [25, 35]. The role of *GauERF105* in the response to *Verticillium dahliae* was verified using VIGS. The silent plants turned yellow, wilted, or even died, compared with the control plants. After silencing the *GauERF105* gene, plants are more sensitive to Verticillium wilt. Cotton disease index survey, lignin staining, *Verticillium dahliae* recovery culture, and quantitative experiment of *Verticillium dahliae* showed that the silencing of the *GauERF105* gene weakens the defense ability of plants against pathogens. Compared with the control, the expressions of *GhPR3*, *GhPR4*, and *GhPR5* were significantly downregulated in the silent plants, which indicated that when the expression of the *GhERF105* gene interfered, the expression of its downstream genes was inhibited, which weakened the plant’s disease resistance. Compared with non-inoculated plants, the expression levels of disease-related protein genes in cotton or *Arabidopsis thaliana* were increased, indicating that the plants activated SA, ET/JA, and other hormone transmission pathways after being subjected to biological stress to participate in the defense response of plants.

Based on these findings, it can be concluded that *GauERF105* functions as a positive regulator of Verticillium wilt resistance in both Arabidopsis and cotton. Overall, our findings provide further evidence of the importance of ERF transcription factors such as *GauERF105* in conferring resistance to fungal pathogens and may have implications for the development of novel strategies for enhancing crop productivity and disease resistance in agriculture.

## Conclusions

The severe impact of *Verticillium dahliae* infection on cotton crops in China has resulted in increasing yield losses each year, highlighting the urgent need for disease-resistant cotton varieties. Identifying candidate genes responsible for disease resistance, particularly against *Verticillium dahliae* in cotton, is crucial for developing such varieties. In this study, we selected and screened the *GauERF105* gene from *Gossypium Australe* to investigate its potential role in protecting against *Verticillium dahliae* infection in both cotton and Arabidopsis. Overexpression experiments in Arabidopsis and VIGS in cotton demonstrated that *GauERF105* acts as a positive regulator of disease resistance. Further validation through RT-qPCR, Trypan blue, DAB, and lignin staining confirmed the crucial role of *GauERF105* in enhancing resistance against *Verticillium dahliae* infection in cotton. Our findings suggest that *GauERF105* could be utilized in future breeding programs aimed at developing disease-resistant cotton varieties.

## Methods

### Plant material, *Verticillium dahliae* strains, and gene selection

The Cotton Research Institute of the Chinese Academy of Agricultural Sciences, Anyang, China, supplied the diploid wild cotton (*G. australe*) and the Colombian ecotype Arabidopsis used in this study. The cotton seedlings are grown in a growth box with a light/dark cycle of 16/8 h and a temperature of 27 °C (day)/23 °C (night). The wild-type *Arabidopsis thaliana* (COL-0) was cultured under a light/dark cycle of 16/8 h at a constant temperature of 22 °C. The highly invasive strain of *V. dahliae* (LX2-1) was used for disease resistance identification, and the preparation of conidia suspension (10^7^ conidia mL^−1^ for cotton, 10^6^ and 10^3^ conidia for Arabidopsis) [25]. From previously available RNA-Seq data [25, 36], we performed a transcriptome-wide search to select all the ERF family genes in *Gossypium australe,* and then based on higher expressions in response to *Verticillium dahliae* infection we selected *GauERF105* for further experiments.

### Gene cloning and phylogenetic analysis

The RNA samples were extracted using the RNAprep Pure Plant Plus Kit (TIANGEN BIOTECH, Beijing, China), and their quality was assessed through agarose gel electrophoresis and spectrophotometry. TranScript-All-in-One First-Strand cDNA Synthesis SuperMix (TransGen, Beijing, China) reverse transcription kit was used to obtain cDNA. Primers were designed by using the CDS sequence of *GauERF105*. cDNA of *G. australe* was used as a template, and P505 high-fidelity polymerase (Vazyme, Nanjing, China) was used to amplify the target gene. Amino acid sequences of other cotton ERF members from the NCBI website were downloaded. DNAMAN software was used for multiple sequence alignment, and MEGA-X was used to construct a phylogenetic tree.

### Generation of transgenic *Arabidopsis* lines

The *GauERF105* gene was linked with 'BamHI' and 'SacI' restriction sites using the homologous recombination method. This resulted in the creation of an expression vector named PBI121-*GauERF105*. *Agrobacterium tumefaciens* GV3101 was transformed with this vector. Transgenic *Arabidopsis thaliana* plants were obtained using the flower soaking method [37]. The transgenic lines (T0, T1, and T2 seeds) were screened on half-strength Murashige and Skoog medium with kanamycin added. The T3 transgenic lines were evaluated and the expression was checked by qRT-PCR and then used in subsequent experiments. The 20-day-old Arabidopsis plants were inoculated with *V. dahliae*. Twenty days after inoculation (20Dpi), the symptoms were scored. According to the degree of leaf yellowing, the degree of resistance to VW is graded from 0 to 4. The calculation method of the disease index was kept the same as above.

### Histochemical staining of cotton stem lignin

Cotton samples were subjected to lignin histochemical staining using the Wiesner method [38]. The parts of cotton cotyledon nodes of wild-type, TRV: 00 and TRV: *GhERF105* plants were sectioned by hand. Wiesner reagent [3% (w/v) phloroglucinol in dd solution, solubilized with absolute ethanol] was used to dip the slices for 5 min and wash twice with distilled water. Then, acidify with 6% hydrochloric acid solution for 5 min, and wash away residual after hydrochloric acid treatment. Place the slices on a glass slide to observe and take pictures under a stereo microscope.

### Measurement of antioxidants and oxidants enzyme activities

To evaluate oxidative stress and antioxidant activity, hydrogen peroxide (H_2_O_2_) content was measured using the YX-W-A400 assay kit (Sino Best Biological Technology Co., Ltd). Malondialdehyde (MDA) content was measured as a marker of oxidative damage, following established protocols [39, 40]. Key antioxidant enzymes peroxidase (POD) and superoxide dismutase (SOD) were assayed based on previously described methods [41, 42] to provide insights into antioxidant capacity.

### Fungal recovery assay of cotton stems after *V. dahliae* inoculation

The fungal recovery assay was conducted according to the previously described protocol by [43]. We randomly took cotton plants treated with *Verticillium dahliae*. Stem sections were used that were above the cotyledons, placed in a sterilized triangular flask, and then use disinfectant to disinfect the surface of the cotton stems for 7 min. Stem sections were then sterilized immediately after disinfection with Sodium hypochlorite. Stem sections were washed with ddH_2_O and rinsed 3 times for 5 min. Samples were placed in a petri dish containing PDA with cephalosporin and incubated at 25 °C in the dark for 3–5 days to observe the fungal growth.

### DAB staining

Hydrogen peroxide production and accumulation in the leaves were measured using the 3,3'diaminobiphenyl (DAB) staining method, which was described by [44]. After 72 h of inoculation with *Verticillium dahliae*, Arabidopsis leaves were taken, rinsed with distilled water, and then dried with filter paper. Leaves were added in a 2 mL centrifuge tube, and then an appropriate amount of DAB staining solution for staining, and then placed in the dark at room temperature for 8 h. After 8 h the staining solution was removed and then 95% ethanol was added to remove chlorophyll repeat this step 3 times., Sterilized water was then used to wash the leaves before taking pictures.

### Trypan blue staining

The leaves of Arabidopsis plants transformed with *GauERF105* and the wild-type Arabidopsis control group were inoculated for 72 h and then soaked in a solution of trypan blue dye (10 mL lactic acid, 10 mL glycerin, 10 g phenol, 10 mg trypan blue, and 10 mL distilled water). The samples were then subjected to a boiling water bath for 2 min, followed by cooling and decolorization in chloral hydrate (2.5 g/mL). The decolorizing solution was changed every day for 3 days, and the samples were washed with sterilized water before being photographed.

### Cotton VIGS and quantification of disease resistance

The VIGS procedure used in this study was based on previously described protocols by [25]. A 432 bp *GauERF105* fragment was amplified and inserted between the *BamHI* and *EcoRI* sites of the tobacco Rattle virus (TRV) binary vector pTRV2. The phytoene desaturase (PDS) gene was used as a marker to detect the reliability of silencing. *Verticillium dahliae* was inoculated 21 days after the silencing. These experiments were repeated three times independently, using more than 35 plants for each treatment. At 25dpi, the seedling's symptoms are divided into five levels: 0, 1, 2, 3, and 4 according to the symptoms on the leaves [45]. The calculation of the Disease index (DI) was as follows [36]:

$$\mathbf{D}\mathbf{I}=[(\sum \mathbf{d}\mathbf{i}\mathbf{s}\mathbf{e}\mathbf{a}\mathbf{s}\mathbf{e}\,\mathbf{g}\mathbf{r}\mathbf{a}\mathbf{d}\mathbf{e}\mathbf{s}\times \mathbf{n}\mathbf{u}\mathbf{m}\mathbf{b}\mathbf{e}\mathbf{r}\,\mathbf{o}\mathbf{f}\,\mathbf{i}\mathbf{n}\mathbf{f}\mathbf{e}\mathbf{c}\mathbf{t}\mathbf{e}\mathbf{d}\,\mathbf{p}\mathbf{l}\mathbf{a}\mathbf{n}\mathbf{t}\mathbf{s})/(\mathbf{t}\mathbf{o}\mathbf{t}\mathbf{a}\mathbf{l}\,\mathbf{n}\mathbf{u}\mathbf{m}\mathbf{b}\mathbf{e}\mathbf{r}\,\mathbf{o}\mathbf{f}\,\mathbf{s}\mathbf{c}\mathbf{o}\mathbf{r}\mathbf{e}\mathbf{d}\,\mathbf{p}\mathbf{l}\mathbf{a}\mathbf{n}\mathbf{t}\mathbf{s}\times 4)]\times 100.$$

### Expression analysis of defense marker genes

Leaf tissues of cotton and Arabidopsis thaliana, which were inoculated with *Verticillium dahliae*, were collected at 48 and 72 h post-infection, respectively. Total RNA was extracted from the frozen tissues using liquid nitrogen. The expression levels of defense marker genes in cotton and Arabidopsis were determined using specific primers for disease-related proteins (PRs), as previously described by [24]. The experiments were conducted with three biological replicates and three technical replicates.

### Expression analysisvia* qRT-PCR*

Total RNA was extracted and reverse-transcribed into cDNA. For PCR amplification SYBR®qPCR Master Mix (Vazyme, Nanjing, China) was used. The cotton *GhUBQ7* gene and the Arabidopsis *AtActin* gene were used as internal reference genes for qRT-PCR analysis. The 2^−ΔΔct^ method was used to calculate the relative expression of genes. The qRT-PCR assays were performed as described previously [36]. For *GauERF105* following promers were used “F = AGAGTGAGCCTGTTGGAGTC”, “R = TACATAACCTCAAGCCTGGAGTA”.

## Data Availability

Not applicable.
